# Unraveling monoclonal gammopathy of renal significance: a mini review on kidney complications and clinical insights

**DOI:** 10.3389/fneph.2024.1439288

**Published:** 2024-09-12

**Authors:** Mythri Shankar, Manjusha Yadla

**Affiliations:** ^1^ Department of Nephrology, Institute of Nephrourology, Bengaluru, India; ^2^ Department of Nephrology, Gandhi Medical College, Hyderabad, India

**Keywords:** monoclonal, gammopathy, renal, significance, pathogenesis, treatment, kidney, plasma cells

## Abstract

Monoclonal gammopathy of renal significance (MGRS) is where kidney injury occurs due to the accumulation or effects of abnormal monoclonal proteins. These proteins, originating from non-cancerous or pre-cancerous plasma cells or B cells, deposit in specific areas of the kidney. Mechanisms contributing to MGRS include high levels of vascular endothelial growth factor secretion, autoantibodies targeting complement components, and targeting specific receptors leading to nephropathy. Kidney lesions in monoclonal gammopathy of renal significance (MGRS) are classified based on the presence of organized or nonorganized deposits, including fibrillar, microtubular, or crystal inclusions. Kidney biopsy is essential for confirming the diagnosis of MGRS by identifying monoclonal immunoglobulin deposits. Immunofluorescence helps determine the class of light and/or heavy chain involved in MGRS. The treatment approach is clone-directed and hence it depends on the presence of B cell clone or plasma cell clone or any detectable monoclonal protein. Chemotherapy targeting plasma cell or B cell malignancies and autologous hematopoietic cell transplantation may be used to manage MGRS. Kidney outcomes in MGRS patients strongly correlate with the hematologic response to chemotherapy.

## Introduction

Circulating monoclonal protein (M protein) is seen in 3% of individuals older than 50 years. This percentage increases to 5% after age 70 years (1, 2). Such individuals are diagnosed with a condition known as monoclonal gammopathy of undetermined significance (MGUS). MGUS is a condition where M-protein is below 30 g/l and a bone marrow examination shows less than 10% monoclonal plasma cells, along with the absence of damage to vital organs or events associated with multiple myeloma. It is important to note that MGUS is an asymptomatic condition. However, in some instances, monoclonal gammopathy can lead to severe organ damage, but it doesn’t meet the criteria for overt multiple myeloma (MM) or other malignant lymphoproliferative disorders such as B-cell non-Hodgkin lymphoma, which includes Waldenström’s macroglobulinemia (WM) or chronic lymphocytic leukemia (CLL) (3).

Hence, the International Kidney and Monoclonal Gammopathy Research Group (IKMG), in the year 2012, coined the term “monoclonal gammopathy of renal significance” (MGRS) to characterize a range of kidney disorders caused by the secretion of M-protein from a clone of B-cells, lymphoplasmacytic cells, or plasma cells that fail to satisfy current hematologic criteria for specific treatment. Recently, the IKMG expanded the definition of MGRS to encompass all B-cell or plasma cell proliferative disorders that generate a kidney-damaging M-protein, including conditions like smoldering multiple myeloma (MM), smoldering Waldenström’s macroglobulinemia (WM), and monoclonal B-cell lymphocytosis (MBL). Additionally, low-grade B-cell lymphomas and low-grade chronic lymphocytic leukemia (CLL) associated with kidney problems are now considered part of the updated MGRS definition. These clones, responsible for secreting M-proteins, can have a direct nephrotoxic effect and can also indirectly harm the kidneys through complement activation. Furthermore, these small B-cell or plasma cell clones can affect organs, such as the skin and peripheral nerves, leading to introduction of the concept of “monoclonal gammopathy of clinical significance” (MGCS) (4).

**Figure 1:**
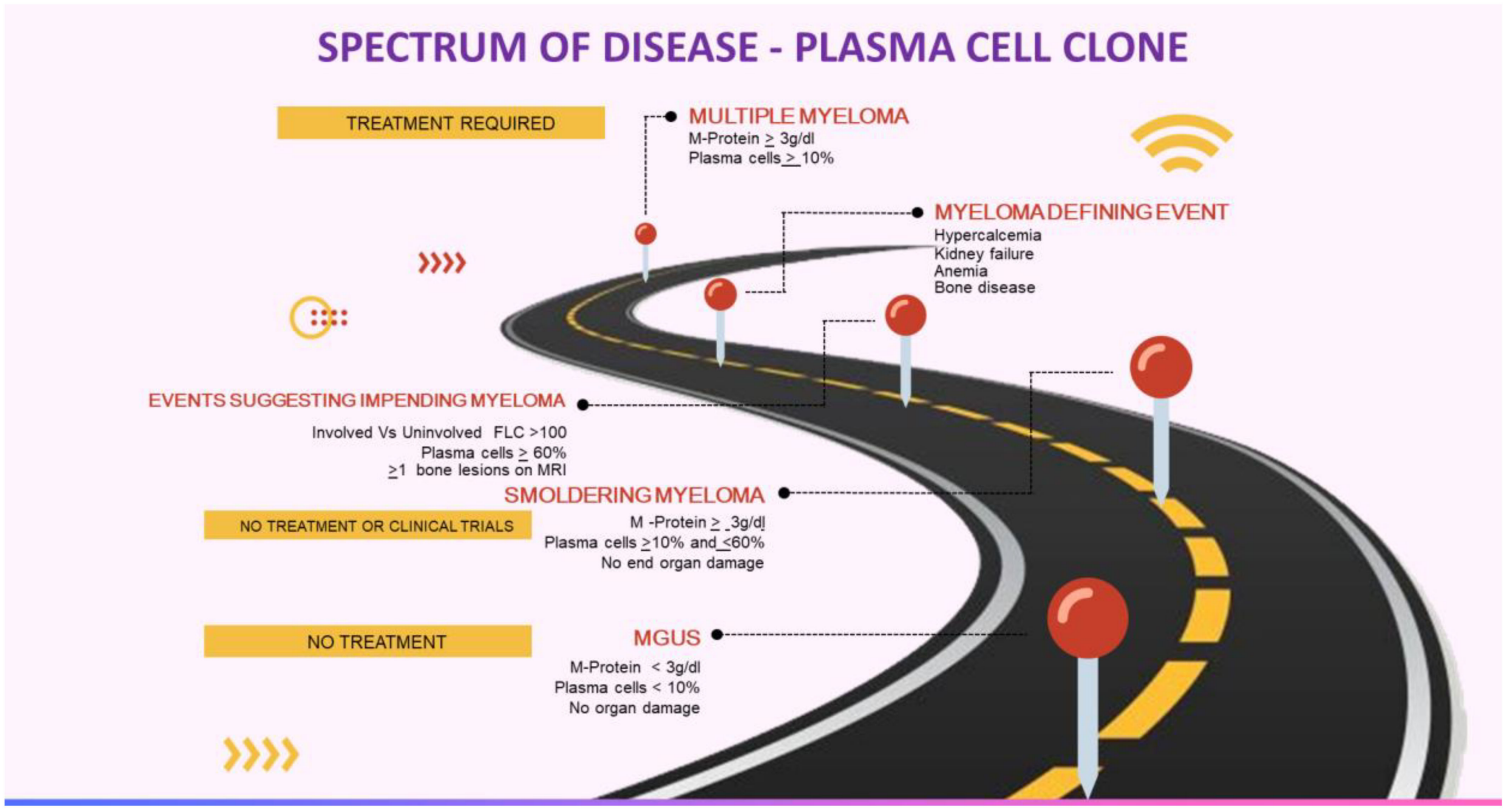
Multiple myeloma continuum. Courtesy: Mythri Shankar.

Anti-tumor therapy is currently not recommended for MGUS, smoldering myeloma, or asymptomatic WM (**Table 1**, **Figure 1**). The patients are closely monitored and treatment is considered only if they develop any symptoms or at the risk of developing any symptoms. However, this approach is not valid in MGRS as the M-protein may still cause kidney damage even if the tumor burden is not high. In such instances, the monoclonal gammopathy has a known significance due to the presence of kidney disease, which carries an increased risk of progressing to end-stage kidney disease (ESKD). Furthermore, there have been reported cases of recurrence after kidney transplantation (5–12).

**Table 1:** Definitions of B-cell and plasma cell proliferative disorders.

Plasma cell disorder	Criteria
Multiple myeloma	The presence of 10% or more clonal plasma cells in the bone marrow or a biopsy-confirmed bony or extramedullary plasmacytoma, along with one or more of the following criteria for myeloma: - Hypercalcemia: Serum calcium level > the upper normal limit by 0.25 mmol/L - Renal insufficiency: Creatinine clearance < 40 mL per minute or serum creatinine > 177 µmol/L - Anemia: Hemoglobin level ≤ 6.2 mmol/L - Bone lesions: One or more lesions visible on skeletal radiography, CT, or PET-CT Additionally, the diagnosis can be supported by any of these biomarkers indicating disease progression: - More than 60% clonal bone marrow plasma cells - Serum free light chain ratio of more than 100:1 between involved and uninvolved chains - Focal lesions on MRI scans
Smoldering multiple myeloma	Serum M-protein levels >30 g/L or urinary M-protein of 500 mg over 24 hours, and/or clonal bone marrow plasma cells between 10-60%. - No presence of SLIM-CRAB* symptoms or amyloidosis. *In 2014, the International Myeloma Working Group (IMWG) classified patients with smoldering multiple myeloma (SMM) into the diagnostic category of multiple myeloma (MM) based on biomarker criteria. This classification includes individuals with 60% or more bone marrow plasma cells (BMPCs), a free light chain ratio (FLCratio) of 100 or greater, and one or more MRI-defined focal lesions measuring 5 mm or more, collectively referred to as SLiM CRAB MM.

## Pathogenesis

In MGRS, kidney damage primarily occurs due to the abnormal buildup or impact of monoclonal proteins in the kidney. These proteins, which may be light chains, heavy chains, or entire immunoglobulins, are produced by small, benign or pre-malignant clones of plasma cells or B cells. The specific locations within the kidney—such as the glomeruli, tubules, vessels, or interstitial areas—where these monoclonal proteins accumulate vary according to the unique biochemical characteristics of the involved pathogenic light and/or heavy chains.

Moreover, other mechanisms that contribute to the development of MGRS have also been identified, in addition to the deposition of monoclonal proteins within the kidney:

- vascular endothelial growth factor (VEGF) secretion in high amounts is implicated in patients with POEMS syndrome—characterized by polyneuropathy, organomegaly, endocrinopathy, monoclonal gammopathy, and skin changes. This includes conditions like membranoproliferative glomerulonephritis-like lesions, thrombotic microangiopathy, and mesangiolysis with the formation of microcapillaries (13).
- Monoclonal proteins may function as C3 nephritic factor or autoantibodies targeting complement components such as complement factor H, factor I, and complement receptor 1 (CR1). This results in uncontrolled activation of the alternative complement pathway, causing C3 glomerulopathy linked with monoclonal gammopathy. Both processes facilitate the accumulation of complement factors such as C3 in the kidney, without substantial deposits of immunoglobulin (14).
- Circulating monoclonal immunoglobulin autoantibodies may target the phospholipase A2 receptor, leading to a type of membranous nephropathy that often recurs quickly after kidney transplantation (15). Additionally, there have been reports of monoclonal anti-glomerular basement membrane (GBM) disease caused by circulating monoclonal antibodies that attack type IV collagen (16).

## Classification according to pathology

The kidney lesions linked to MGRS can be classified based on the ultrastructural features of any deposits found in the kidney (17). These deposits can be categorized as either organized (i.e., having a substructure) or nonorganized (i.e., granular and lacking substructure). Occasionally, deposits may not be visible within the kidney.

Organized deposits in MGRS lesions are further classified as microtubular deposits, fibrillar deposits or crystal inclusions.

## Fibrillar deposits

These include amyloidosis and fibrillary GN. In amyloidosis, the fibrillar deposits are Congo red positive, while in fibrillary glomerulonephritis, it is usually negative. Staining for the DnaJ heat shock protein family (Hsp40) member B9 (DNAJB9) helps differentiate between fibrillary glomerulonephritis (positive) and amyloid (negative) (18). However, recent reports suggest that fibrillary glomerulonephritis could be an independent entity unrelated to monoclonal gammopathy (19).

## Microtubular deposits

Lesions in MGRS with microtubular deposits are seen in conditions like monotypic immunotactoid glomerulopathy and monoclonal (type 1 and some type 2) cryoglobulinemia. Microtubules differ from fibrils because they are usually larger and feature hollow centers (20).

## Crystal inclusions

Lesions with crystal inclusions cover disorders like light chain proximal tubulopathy. It is a condition where the light chain crystals deposit in proximal tubular epithelial cells. Rarely, a non-crystalline deposit variant of proximal tubulopathy is also seen. Light chain crystalline podocytopathy features crystalline inclusions primarily within podocytes but also in other kidney cell types like proximal and distal tubular cells, endothelial cells, interstitial histiocytes, and mesangial cells (21). In crystal-storing histiocytosis, intracytoplasmic light chain crystalline inclusions are found within interstitial histiocytes, and sometimes in proximal tubular cells and podocytes (22). Cryocrystalglobulinemia is characterized by deposits made up of entire monoclonal immunoglobulins within glomerular endothelial cells or in the subendothelial space or in the vascular lumens or mesangial cells. Extrarenal deposits are also observed in all three conditions (23).

**Figure 2:**
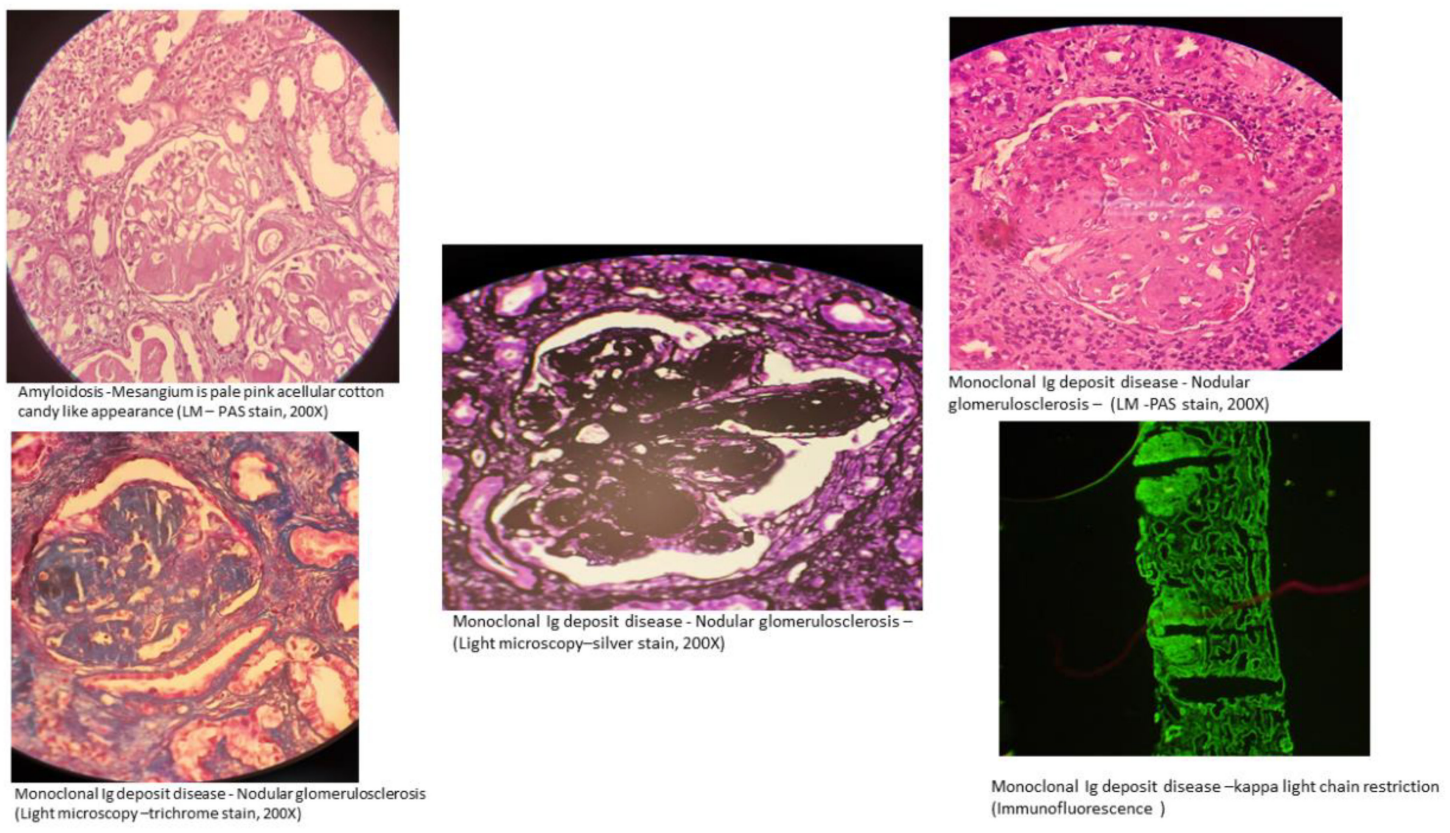
Kidney biopsy images of MGRS. Image Credit: Vinay .KS

Nonorganized deposits in MGRS lesions include conditions classified under monoclonal immunoglobulin deposition diseases (MIDDs), such as light chain, heavy chain, or both light and heavy chain deposition disease, and monoclonal gammopathy-associated proliferative glomerulonephritis (24). This includes diseases involving deposits that are monoclonal immunoglobulin G (IgG), and less commonly, immunoglobulin M (IgM), immunoglobulin A (IgA) or light chain-only. Membranoproliferative glomerulonephritis is the most frequent type of injury observed in monoclonal gammopathy-associated proliferative glomerulonephritis, which encompasses conditions like proliferative glomerulonephritis with monoclonal immunoglobulin deposits (PGNMID) (25, 26) and C3 glomerulopathy with monoclonal gammopathy. In C3 glomerulopathy, the deposits mainly consist of C3 complement (27, 28). This category also includes rare cases of MGRS that histologically resemble polyclonal immunoglobulin-mediated kidney diseases, such as membranous nephropathy and anti-glomerular basement membrane (GBM) antibody (Goodpasture’s) disease, which show monotypic staining during immunofluorescence microscopy (15) (**Figure 2**).

### No deposits

MGRS lesions without any deposits encompass conditions such as thrombotic microangiopathy linked to monoclonal gammopathy and POEMS syndrome (13, 29).

## Limitations of the classification system

Although it offers a structured approach to diagnosing kidney diseases associated with MGRS, it does not offer details regarding the clinical progression or prognosis of these various conditions.

## Clinical manifestations

Kidney disease in patients with MGRS may arise as a complication of already identified premalignant hematologic disorder or non-malignant hematologic disorder, such as MGUS or smoldering multiple myeloma, or it may be the first sign of a monoclonal gammopathy. Similar to the kidney diseases seen in multiple myeloma (30) and other malignant monoclonal gammopathies, those related to MGRS can manifest as acute or subacute kidney injury, chronic kidney disease, proteinuria and/or nephrotic syndrome, or electrolyte imbalances (31). The most common initial symptoms include impaired kidney function and proteinuria, possibly accompanied by hematuria. Additionally, MGRS can mimic kidney diseases typically not associated with monoclonal gammopathies, like membranous nephropathy and anti-glomerular basement membrane (GBM) antibody (Goodpasture’s) disease (15, 32).

### When to suspect MGRS

The diagnosis of MGRS should be considered under the following circumstances:

Indicators associated with MGRS include proteinuria ≥1.5 g/day, hematuria, and an abnormal serum-free light chain ratio (33).

The suspicion should also arise in all patients with a premalignant or non-malignant monoclonal gammopathy with unexplained kidney function impairment and/or proteinuria. Additionally, patients presenting with unexplained kidney dysfunction and/or proteinuria, who upon further investigation with serum or urine protein electrophoresis, immunofixation, or a serum free light chain assay is found to have a monoclonal gammopathy should be suspected of MGRS. It is well established that urine-free light chain assay is not a clinically significant test at this point in time.

## Establishing the diagnosis

In most cases where MGRS is suspected, a kidney biopsy is performed unless there are reasons not to proceed. Confirmation of diagnosis of MGRS is by identifying monoclonal immunoglobulin deposits in the kidney via biopsy, which are characteristically restricted to a particular class of heavy chain or light chain as determined by immunofluorescence. The definitive method to demonstrate the nephrotoxic effects of monoclonal proteins is kidney biopsy, as the identification of monoclonal protein in serum or urine alone does not confirm its role in kidney disease. However, a kidney biopsy may be postponed under certain clinical situations:

In some cases of C3 glomerulonephritis, standard immunofluorescence might not reveal monoclonal immunoglobulin deposits. Here, protease digestion followed by paraffin immunofluorescence is the recommended method to unmask any hidden immunoglobulin deposits. Missing these “masked” monoclonal immunoglobulins can lead to a misdiagnosis, only identifying C3 glomerulonephritis instead of MGRS (33, 34). If no monoclonal immunoglobulins are detectable by paraffin immunofluorescence, the confirmation of circulating monoclonal protein through serum or urine protein electrophoresis, immunofixation, and/or serum free light chain assay is required to diagnose C3 glomerulopathy due to monoclonal gammopathy. Additional tests should include evaluating the alternative complement pathway, such as assessing C3 nephritic factor, C3 levels, C4 levels, C5b-9 complex and anti-complement factor H autoantibodies.

Interestingly, the majority (70 to 80 percent) of proliferative glomerulonephritis with monoclonal immunoglobulin deposits (PGNMID) may not demonstrate plasma cell or B cell clones in bone marrow examinations. In such cases, the monoclonal protein is found exclusively in the kidney and diagnosis is solely by kidney biopsy.

Patients presenting with albuminuria or nephrotic syndrome who have a confirmed diagnosis of immunoglobulin light chain (AL) amyloidosis from biopsies of non-kidney tissues may receive a presumptive diagnosis of renal AL amyloidosis without requiring a kidney biopsy.

Furthermore, patients diagnosed with monoclonal gammopathy displaying signs of Fanconi syndrome (e.g., subnephrotic-range proteinuria, aminoaciduria, hypophosphatemia, normoglycemic glycosuria, hypouricemia), can be presumptively diagnosed with light chain proximal tubulopathy.

Patients with monoclonal gammopathy can undergo kidney biopsy safely. A study involving 1993 patients who had either native or transplant kidney biopsies showed that the incidence of major hemorrhagic complications was comparable between patients with and without monoclonal gammopathy (35).

### Further evaluation of patients diagnosed with MGRS

For patients diagnosed with MGRS, further assessments aim to characterize the clone to determine the appropriate treatment strategy.

**Figure 3:**
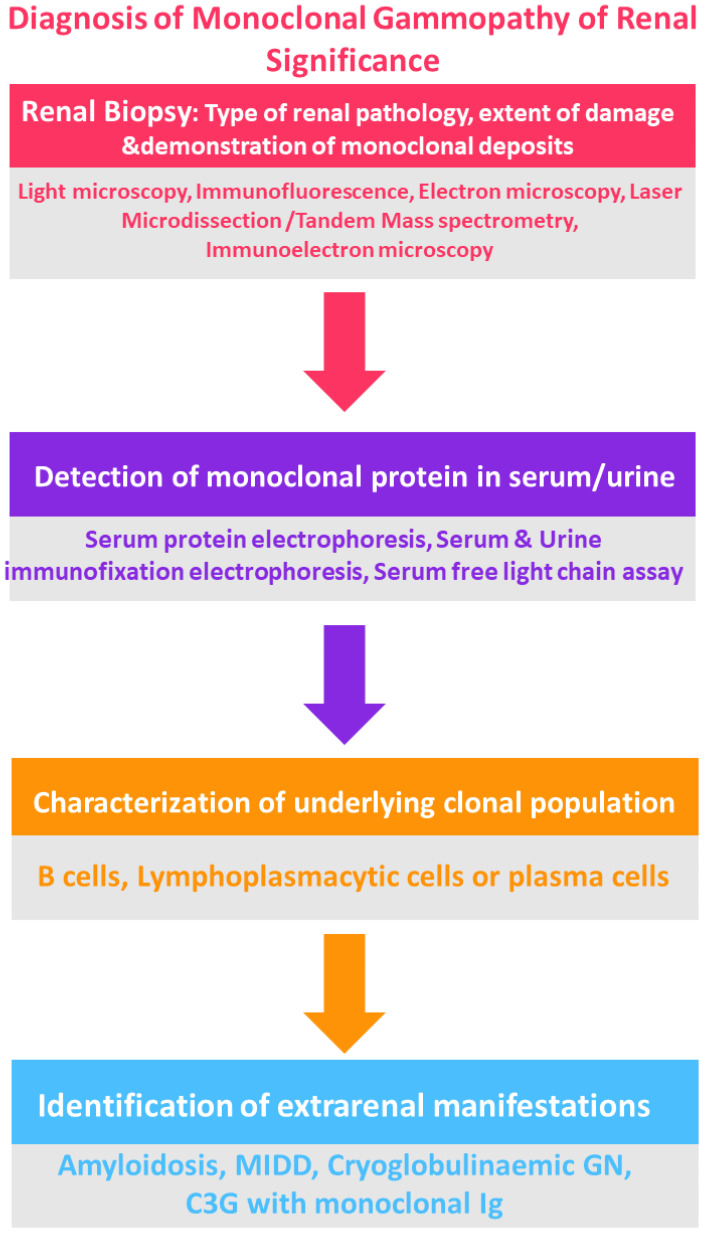
Flowchart for the diagnosis of MGRS. Courtesy: Mythri Shankar.

For patients who either do not show a detectable clone in initial tests, or who possess an IgM monoclonal protein (suggesting a likely B cell or lymphoplasmacytic clone), additional diagnostic measures may include imaging such as computed tomography (CT) of the chest, abdomen, and pelvis, along with positron emission tomography (PET), if available, to identify any B cell clone (25, 36, 37) (**Figure 3**).

## In all cases of MGRS, the following hematologic evaluations are conducted

### Monoclonal protein testing

This includes serum protein electrophoresis and immunofixation, 24-hour urine protein electrophoresis and immunofixation, along with a serum free light chain assay. While the urine-free light chain assay is not recommended due to its lack of any added information, using these tests together improves the sensitivity for detecting monoclonal proteins, particularly in patients with small clones producing minimal protein levels. It is essential that any circulating monoclonal protein discovered matches the type present in kidney deposits. Identifying a serum or urine monoclonal protein is also critical for tracking the effectiveness of treatment.

### Bone marrow aspirate and biopsy

This analysis should encompass immunohistochemistry and flow cytometry to evaluate the surface and intracellular markers of plasma cells and B cells. Staining for kappa and lambda light chains is essential to verify that the identified clone corresponds to the light chain restriction of the monoclonal deposits in the kidney. Additionally, cytogenetic and fluorescence *in situ* hybridization (FISH) analyses are becoming more common in guiding treatment decisions and may prove beneficial in specific scenarios.

For patients without a detectable clone from prior testing, or who possess an IgM monoclonal protein typically produced by B cells, additional evaluations may be necessary. This involves conducting imaging studies such as CT scans of the chest, abdomen, and pelvis, augmented with PET scans when possible, to identify potential B cell clones. Additionally, flow cytometry of peripheral blood lymphocytes is carried out to detect small, low-grade clones like those observed in chronic lymphocytic leukemia and monoclonal B cell lymphocytosis (MBL).

Patients diagnosed with a variant of MGRS that may be associated with extrarenal complications, including AL amyloidosis, monoclonal immunoglobulin deposition disease (MIDD), or monoclonal [type I] cryoglobulinemia, should be specifically assessed for these additional clinical manifestations.

The success in identifying a pathogenic clone in patients with MGRS varies depending on the specific disorder. For instance, in studies of patients with light chain deposition disease, bone marrow biopsy identified a plasma cell clone in 65 to nearly 100 percent of cases (11). In contrast, the detection rate of a clonal source in patients with PGNMID is much lower, ranging from 25 to 30 percent (25). When considering all major case series of MGRS patients, about 40 percent of cases do not have an identifiable clone (38).

## Treatment

**Figure 4:**
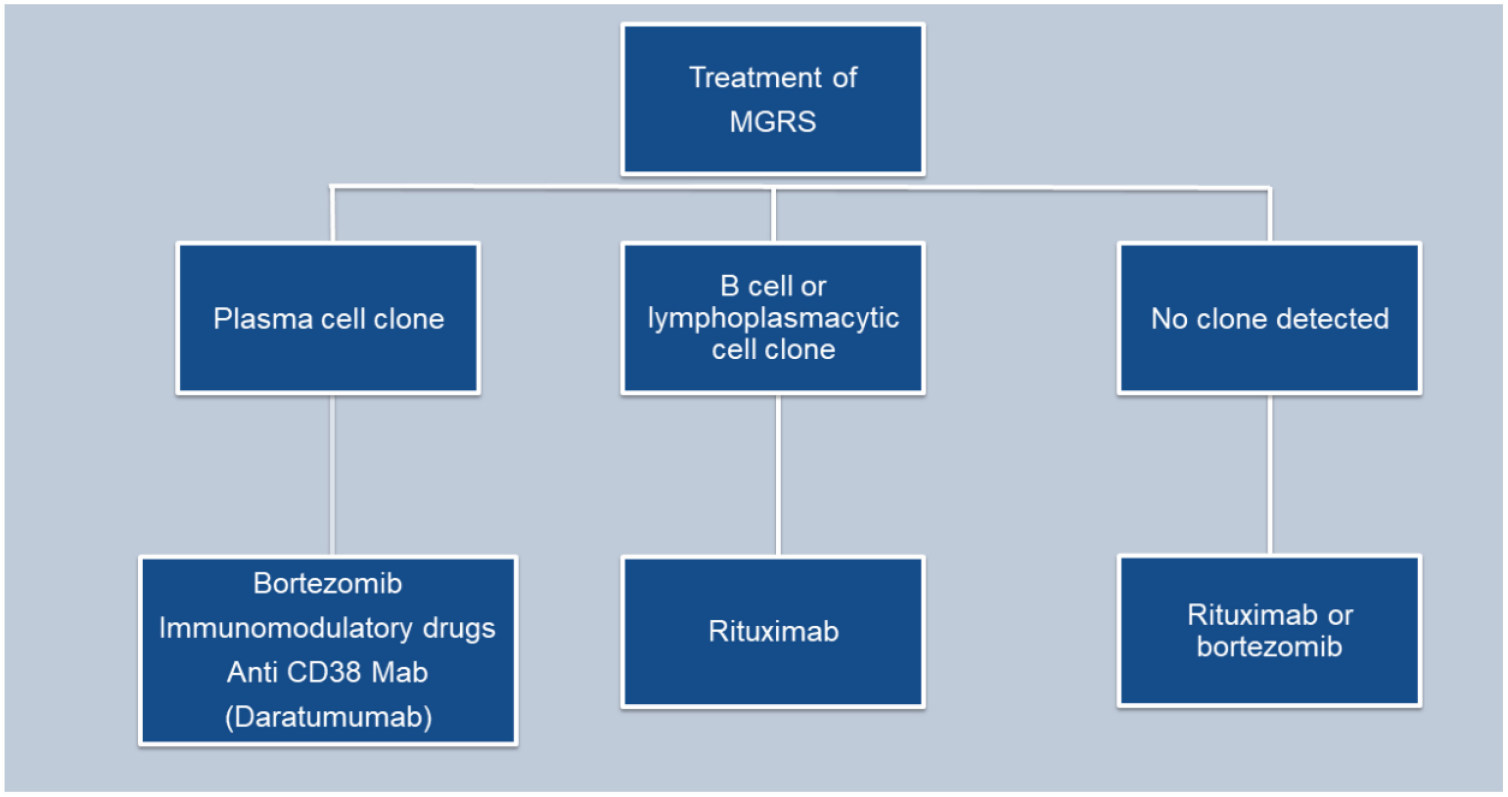
Flowchart depicting the treatment of MGRS.

The main goal in managing monoclonal gammopathy of renal significance (MGRS) is to maintain kidney function and inhibit the progression of any associated extrarenal manifestations. While most MGRS cases result from the deposition of monoclonal immunoglobulins in the kidneys, no treatments are currently available to stop this deposition or remove the already deposited materials (24) (**Figure 4**).

**Figure 5:**
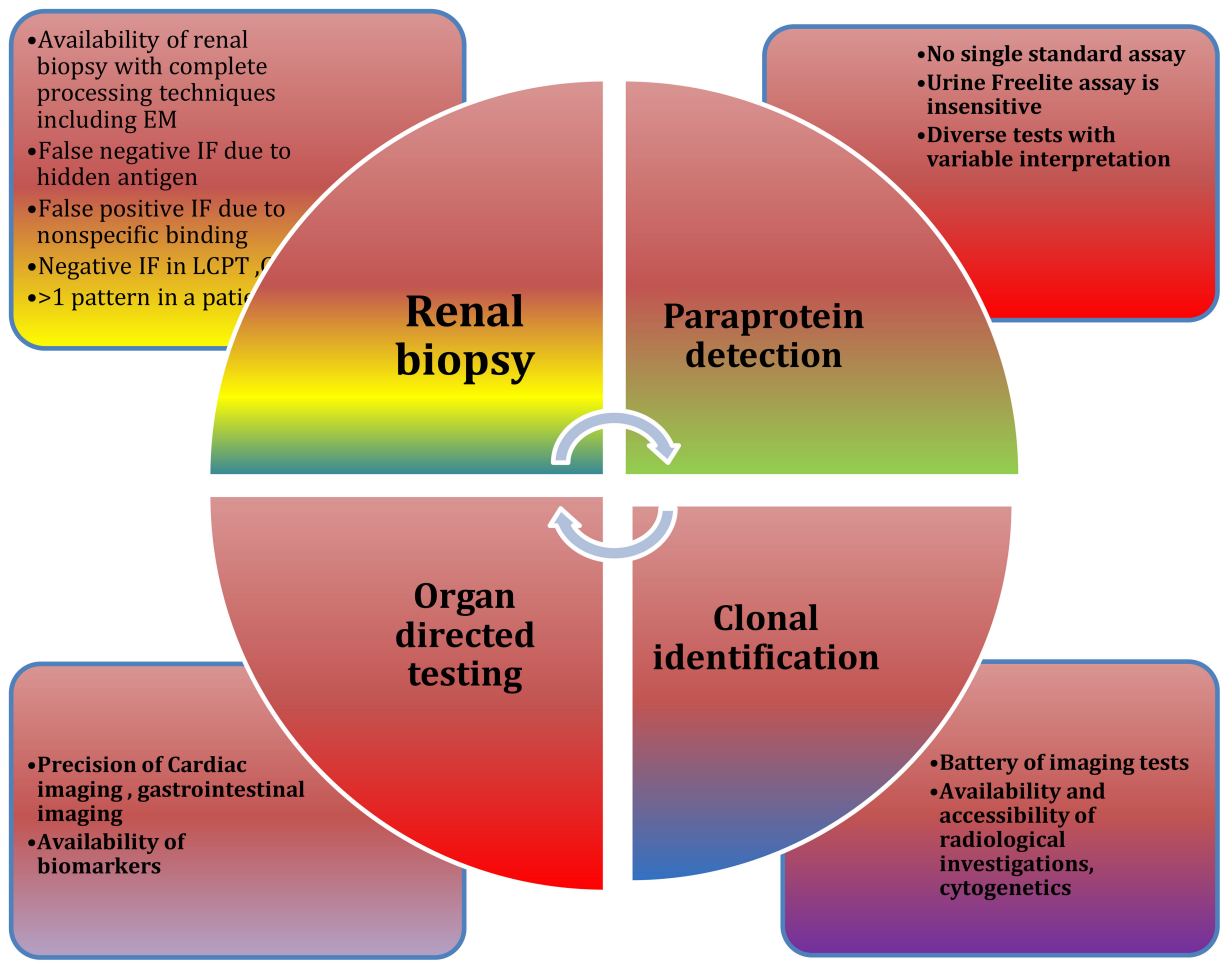
Diagnostic challenges: Challenges are at multiple levels while approaching MGRS.

The treatment strategy for MGRS largely hinges on the type of kidney injury, the characteristics of the clone (be it plasma cell, B cell, or lymphoplasmacytic) that is producing the nephrotoxic monoclonal immunoglobulin, and the ability to reverse or halt further kidney damage. Recent studies indicate that kidney outcomes in MGRS patients correlate strongly with the hematologic response to chemotherapy (39, 40).

Typically, the chemotherapeutic agents used to manage MGRS, target plasma cell or other B cell malignancies. These include proteasome inhibitors like bortezomib, carfilzomib, and ixazomib; monoclonal antibodies such as rituximab and daratumumab; alkylating agents including cyclophosphamide, bendamustine, and melphalan; immunomodulatory drugs like thalidomide, lenalidomide, and pomalidomide; and glucocorticoids such as prednisone and dexamethasone. In cases of conditions such as amyloidosis or monoclonal immunoglobulin deposition disease (MIDD), treatment strategies may include autologous hematopoietic cell transplantation. Preferably, medications that do not necessitate dosage alterations based on kidney function are used to reduce side effects, particularly cytopenias. Treatment decisions for MGRS should be made in association with a hematologist or an oncologist having expertise in using anti-myeloma and anti-lymphoma therapies.

## Treatment of proliferative glomerulonephritis

Patients with PGNMID or C3 glomerulopathy with monoclonal gammopathy, are at an increased risk of progressive kidney disease. To prevent further kidney damage and decline in kidney function, treatment typically centers on eliminating the pathogenic clone responsible for the condition (24) (Tables 2, 3).

The treatment strategy for patients with PGNMID hinges on whether a B cell clone or plasma cell clone or detectable monoclonal protein is present in the urine or serum. The absence of randomized trials means no definitive guide for the optimal treatment approach exists. The support for a clone-directed treatment of PGNMID is largely based on observational studies and one small, uncontrolled trial (38, 41).

For patients with a detectable plasma cell clone, the treatment regimen mirrors that used for multiple myeloma (24). This typically includes a combination of bortezomib, cyclophosphamide and dexamethasone.

Daratumumab is considered an alternative, although data supporting its use are limited (41). Treatment may continue for up to six months if there is evidence of hematologic response and no toxicity.

In patients with an identifiable B cell clone, the treatment protocol mirrors that employed for Waldenström macroglobulinemia. The preferred treatment is rituximab (an anti-CD20 monoclonal antibody), either alone or combined with cyclophosphamide and dexamethasone, or bendamustine, given that most IgM-producing cells are CD20 positive (24).

In patients with PGNMID who do not have a detectable plasma or B cell clone, treatment is determined by the presence of monoclonal protein in the serum or urine. When kidney deposition of monoclonal immunoglobulin matches a detectable monoclonal protein of the same isotype in the serum or urine, it implies a causative relationship. Treatment for these patients involves chemotherapy targeted at eliminating the presumed clone responsible for producing the monoclonal protein. The specific chemotherapy regimen selected depends on the isotype of the monoclonal immunoglobulin identified in the serum or urine, given the absence of a detectable clone.

In patients with non-IgM monoclonal proteins (such as IgG or IgA) detected in the serum or urine and kidney, therapy strategy akin to that used for multiple myeloma is used. For IgM monoclonal protein, treatment is akin to Waldenstorm gammaglobulinemia.

With respect to AL Amyloidosis, at the beginning of the 21st century, patient classification was updated based on Mayo Clinic criteria into stages I, II, or III. This classification was determined by the levels of N-terminal prohormone of brain natriuretic peptide (NT-proBNP) and troponin T. Stage I patients had low levels of both markers (below 332 ng/L and 0.035 mg/L, respectively), stage II patients had high levels of one marker, and stage III patients had high levels of both markers (48, 49). Recently, immunoglobulin free light chain (FLC) levels have been added to these criteria, with minor adjustments to the cut-off points and the inclusion of FLC burden. The main objective of therapy is to achieve the best and most durable hematologic response, tailored to the often delicate condition of the patients. For those with severe cardiac disease, a rapid response is essential due to the direct myocardial toxicity of amyloidogenic light chains, making the quick suppression of FLC a crucial prognostic factor, especially in patients with stage III cardiac disease.

## Current treatment guidelines are as follows

- For patients in stages I and II, the initial treatment should be melphalan combined with dexamethasone (M-Dex). Adding bortezomib to this regimen is expected to enhance hematologic and organ response rates. bortezomib should be introduced rapidly after 1 or 2 courses of M-Dex if there is no clonal response. For patients with advanced chronic kidney disease (CKD), cyclophosphamide is preferred over melphalan, and regimens such as cyclophosphamide-bortezomib-dexamethasone (CBD, also known as CyBorD or VCD) have shown effectiveness. Alternatively, thalidomide can replace bortezomib in the CTD regimen.
- Stage III cardiac patients face significant challenges due to poor median survival rates. Promising preliminary results have been observed with the CBD regimen, which appears to significantly reduce early mortality based on small case series. In young, carefully selected patients, cardiac transplantation might be considered, ideally after achieving hematologic remission.
- In selected patients, particularly those in stages I and II, high-dose melphalan and autologous stem cell transplantation (HDM/ASCT) should be considered if there is no severe renal insufficiency or other advanced organ failure (50).

Given the rarity of Monoclonal Immunoglobulin Deposition Disease (MIDD), controlled studies are lacking, and treatment approaches are based on expert consensus. Achieving an optimal hematologic response is crucial, similar to the treatment of AL amyloidosis, as it can lead to the regression of monoclonal immunoglobulin deposits if complete and sustained remission is achieved (51).

Small retrospective studies have shown that high-dose melphalan combined with autologous stem cell transplantation (HDM/ASCT) is an effective treatment, offering high hematologic response rates and low mortality related to the treatment. This is in contrast to AL amyloidosis, where patients often experience more systemic complications, leading to higher treatment-related mortality. Most data on HDM/ASCT predate the introduction of newer antimyeloma drugs. Initial results suggest that bortezomib-based treatments may yield similar hematologic response rates to HDM/ASCT, akin to treatments for multiple myeloma (52–55).

## Treatment recommendations should be tailored based on the level of kidney impairment

- For patients with CKD stages 1 to 3, the primary goal is to protect kidney function. A bortezomib-based regimen, such as cyclophosphamide, bortezomib, and dexamethasone (CBD), is recommended as the first line of treatment. HDM/ASCT should be considered for selected patients with good overall health and no significant extrarenal issues, particularly if only a partial hematologic response is achieved with the initial treatment.
- For patients with CKD stages 4 and 5, the chances of renal recovery are minimal. For those not eligible for kidney transplantation, the main goal is to preserve the function of other organs, especially the heart. A bortezomib-based regimen like CBD is recommended. If kidney transplantation is planned, the treatment goal is to ensure long-term function of the transplant, which requires an optimal clonal response. In such cases, HDM/ASCT should be considered after 3 to 4 cycles of a CBD-like regimen.

In MGRS-Amyloidosis, a poor prognosis was linked to high creatinine levels, elevated beta-2-microglobulin, and the need for hemodialysis at the time of diagnosis. In MGRS-Non Amyloidosis, the only factor associated with a higher risk of death was being over the age of 65 (56).

Diagnostic challenges at multiple levels are depicted in **Figure 5**. Emerging therapies for MGRS are show in Tables 4.

**Table 2:** Treatment of PGNMID.

Study, Author, Year	Type of study	Type and No of patients	Rx strategy	NR/PR/CR	ESRD
Nasr/2004 (26)	Cohort	Non directed therapy	Steroid, MMF, CTX Cyclophosphamide with Rituximab	-/-/4	3
Gumber et al., 2018 (38)	Retrospective	Clone directed therapy (16)	Rituximab, Cyclophosphamide BORTEZOMIB, GLUCOCORTICOIDS	0/7/6	3 patients who did not receive Rx
Zand et al., 2021 (41)	RCT	Clone directed therapy (11)	Daramtumumab	0/6/4	One patient relapse SAE noted
Zhou et al., 2022 (42)	Retrospective	64 patients	Steroid alone Bortezomib or Rituximab Thalidomide/Lenalidomide + steroid	CR: 7% CR:53% CR:50%	SAE:23% SAE:60% SAE:46%
Lin et al., 2022 (43)	Meta analysis	Targeted therapy: 40 Non targeted therapy: 35	Single/combination drugs No treatment		Efficacy of targeted Rx: 10. X non targeted group

**Table 3:** Treatment of C3glomerulopathy.

Study, Author, Year	Type	No of patients	Treatment strategy	NR/PR/CR	ESRD
Chauvet S et al., 2017 (27)	Retrospective	50: 29 Targeted Rx 21 unRx	22 patients: bortezomib 5 patients:cyclophosphamide or melphalan, 2 patients: rituximab	Higher rate of response Higher survival	
Ravindran et al., 2018 (44)	Case series	36: 16 Targeted Rx 17 non targeted Rx		Stable renal function in 44% CR/PR in 41%	Though similar response in untargeted and targeted group, targeted had more severe disease
MGRS in Light chain proximal tubulopathy					
Ma CX et al., 2004 (45)	Retrospective	38 patients 7 patients: No Rx	Antimyeloma drugs	Stable kidney function	3/7 untreated:ESRD
Stokes MB et al., 2016 (46)	Case series	27 patients 9: No Rx	Chemotherapy		29% untreated: ESRD
Light chain crystalline podocytopathy					
Nasr et al., 2023 (47)	Case series	25 patients	21: Anti plasma cell Rx 6: ASCT		6 patients: ESRD

**Table 4:** Newer therapies in management of AL Amyloidosis appear to be promising, offering the advantages of improved organ response and the overall survival rate.

Study	Ongoing/completed	Drug	Disease	Outcome/status
ANDROMEDA (57)	Completed	Daratumumab	AL Amyloidosis	Better overall organ response
Danai Dama et al. (58)	Completed	Venetoclax	AL Amyloidosis	Hematological response - 97%, VGPR -91%, cardiac response- 74% renal response in 46%
NEOD001(VITAL) (59)	Completed	Biratimimab	AL Amyloidosis	Poor outcomes compared to placebo
Gertz et al. (VITAL) (60)	Completed	Biratimimab +Standard of care for AL	AL Amyloidosis	*Post hoc* analysis: Better survival rates
AFFIRM -AL (61)	Ongoing	Biratimimab +Standard of care in Mayo -IV AL	AL Amyloidosis	

### Monitoring the response to therapy

The following tests are conducted monthly to assess together the hematologic and kidney responses to treatment:

- Serum protein electrophoresis and immunofixation.
- 24-hour urine collection for total protein, protein electrophoresis, and immunofixation.
- Serum free light chain assay.
- Serum creatinine.

Evaluations can be conducted every two to three months for patients who have finished active treatment. Nevertheless, a substantial portion of MGRS patients, especially those with PGNMID, initially may not exhibit detectable circulating monoclonal proteins. In these cases, assessing a hematologic response isn’t feasible, and monitoring is often limited to serum creatinine levels and proteinuria quantification. Despite this, we should continue to track monoclonal protein levels as initially outlined since these proteins may become detectable later in the disease’s progression (62, 63).

## Conclusion

Here is a conclusion for the document:

In summary, monoclonal gammopathy of renal significance (MGRS) encompasses a diverse array of kidney disorders driven by monoclonal proteins. The diagnostic and treatment approaches for MGRS must be tailored to the specific type of kidney damage and the characteristics of the pathogenic monoclonal proteins involved. Effective management relies on early detection, precise characterization of the monoclonal protein, and targeted therapy aimed at reducing the production of these proteins. Despite advances in treatment strategies, including the use of bortezomib-based regimens and autologous hematopoietic cell transplantation, the prognosis of MGRS remains variable. Continued research and clinical trials are essential to improve outcomes and develop more effective treatments for patients affected by this complex condition.

## References

[1] Rajkumar SV Dimopoulos MA Palumbo A. International Myeloma Working Group updated criteria for the diagnosis of multiple myeloma. Lancet Oncol. (2014) 15:548. 10.1016/S1470-2045(14)70442-5 25439696

[2] Kyle RA Therneau TM Rajkumar SV Larson DR Plevak MF Offord JR. Prevalence of monoclonal gammopathy of undetermined significance. N Engl J Med. (2006) 354:1362–9. 10.1056/NEJMoa054494 16571879

[3] van de Donk NW Palumbo A Johnsen HE Engelhardt M Gay F Gregersen H. The clinical relevance and management of monoclonal gammopathy of undetermined significance and related disorders: recommendations from the European Myeloma Network. Haematologica. (2014) 99:984–96. 10.3324/haematol.2013.100552 PMC4040895 24658815

[4] Fermand JP Bridoux F Dispenzieri A Jaccard A Kyle RA Leung N. Monoclonal gammopathy of clinical significance: a novel concept with therapeutic implications. Blood. (2018) 132:1478–85. 10.1182/blood-2018-04-839480 30012636

[5] Angioi A Amer H Fervenza FC Sethi S. Recurrent light chain proximal tubulopathy in a kidney allograft. Am J Kidney Dis. (2016) 68:483–7. 10.1053/j.ajkd.2016.04.021 27321964

[6] Roth RM Benson D Hebert LA Bissell MG Satoskar AA Nadasdy T. Progressive renal light chain amyloidosis with the absence of detectable free monoclonal light chains after an autologous hematopoietic stem cell transplant for amyloid light chain amyloidosis. Arch Pathol Lab Med. (2013) 137:1304–8. 10.5858/arpa.2012-0159-CR 23991744

[7] Miyazaki D Yazaki M Gono T Kametani F Tsuchiya A Matsuda M. AH amyloidosis associated with an immunoglobulin heavy chain variable region (VH1) fragment: a case report. Amyloid. (2008) 15:125–8. 10.1080/13506120802006229 18484339

[8] Mallett A Tang W Hart G McDonald SP Hawley CM Badve SV. End-stage kidney disease due to fibrillary glomerulonephritis and immunotactoid glomerulopathy - outcomes in 66 consecutive ANZDATA registry cases. Am J Nephrol. (2015) 42:177–84. 10.1159/000440815 26418732

[9] Sathyan S Khan FN Ranga KV. A case of recurrent immunotactoid glomerulopathy in an allograft treated with rituximab. Transplant Proc. (2009) 41:3953–5. 10.1016/j.transproceed.2009.03.100 19917422

[10] Neel A Perrin F Decaux O Dejoie T Tessoulin B Halliez M. Long-term outcome of monoclonal (type 1) cryoglobulinemia. Am J Hematol. (2014) 89:156–61. 10.1002/ajh.23608 24532335

[11] Sayed RH Wechalekar AD Gilbertson JA Bass P Mahmood S Sachchithanantham S. Natural history and outcome of light chain deposition disease. Blood. (2015) 126:2805–10. doi: 10.1182/blood-2015-07-658872 PMC4732758 26392598

[12] Nambirajan A Bhowmik D Singh G Agarwal SK Dinda AK. Monoclonal gammopathy of renal significance with light chain deposition disease diagnosed postrenal transplant: a diagnostic and therapeutic challenge. Transpl Int. (2015) 28:375–9. 10.1111/tri.12497 25441103

[13] Sanada S Ookawara S Karube H Shindo T Goto T Nakamichi T. Marked recovery of severe renal lesions in POEMS syndrome with high-dose melphalan therapy supported by autologous blood stem cell transplantation. Am J Kidney Dis. (2006) 47:672–9. doi: 10.1053/j.ajkd.2006.01.004 16564945

[14] Jokiranta TS Solomon A Pangburn MK Zipfel PF Meri S. Nephritogenic lambda light chain dimer: a unique human miniautoantibody against complement factor H. J Immunol. (1999) 163:4590–6. 10510403

[15] Debiec H Hanoy M Francois A Guerrot D Ferlicot S Johanet C. Recurrent membranous nephropathy in an allograft caused by IgG3κ targeting the PLA2 receptor. J Am Soc Nephrol. (2012) 23:1949–54. doi: 10.1681/ASN.2012060577 PMC3507371 23123401

[16] Tsuji T Ohashi N Sato T Goto D Nagata S Matsuyama T. Monoclonal immunoglobulin G1 κ-type atypical antiglomerular basement membrane disease accompanied by necrotizing glomerulonephritis. Clin Nephrol. (2020) 93:152–7. doi: 10.5414/CN109889 31854296

[17] Leung N Bridoux F Batuman V Chaidos A Cockwell P D’Agati VD. The evaluation of monoclonal gammopathy of renal significance: a consensus report of the International Kidney and Monoclonal Gammopathy Research Group. Nat Rev Nephrol. (2019) 15:45–59. doi: 10.1038/s41581-018-0077-4 30510265 PMC7136169

[18] Alexander MP Dasari S Vrana JA Riopel J Valeri AM Markowitz GS. Congophilic fibrillary glomerulonephritis: A case series. Am J Kidney Dis. (2018) 72:325–36. doi: 10.1053/j.ajkd.2018.03.017 29866458

[19] Andeen NK Kung VL Robertson J Gurley SB Avasare RS Sitaraman S. Fibrillary glomerulonephritis, DNAJB9, and the unfolded protein response. Glomerular Dis. (2022) 2:164–75. doi: 10.1159/000525542 PMC9936766 36817290

[20] Terrier B Karras A Kahn JE Le Guenno G Marie I Benarous L. The spectrum of type I cryoglobulinemia vasculitis: new insights based on 64 cases. Med (Baltimore). (2013) 92:61–8. doi: 10.1097/MD.0b013e318288925c PMC4553985 23429354

[21] Larsen CP Bell JM Harris AA Messias NC Wang YH Walker PD. The morphologic spectrum and clinical significance of light chain proximal tubulopathy with and without crystal formation. Mod Pathol. (2011) 24:1462–9. doi: 10.1038/modpathol.2011.104 21701535

[22] El Hamel C Thierry A Trouillas P Bridoux F Carrion C Quellard N. Crystal-storing histiocytosis with renal Fanconi syndrome: pathological and molecular characteristics compared with classical myeloma-associated Fanconi syndrome. Nephrol Dial Transplant. (2010) 25:2982–90. doi: 10.1093/ndt/gfq129 20356978

[23] Leung N Buadi FK Song KW Magil AB Cornell LD. A case of bilateral renal arterial thrombosis associated with cryocrystalglobulinaemia. NDT Plus. (2010) 3:74–7. doi: 10.1093/ndtplus/sfp140 PMC4421549 25949411

[24] Sethi S Rajkumar SV. Monoclonal gammopathy-associated proliferative glomerulonephritis. Mayo Clin Proc. (2013) 88:1284–93. doi: 10.1016/j.mayocp.2013.08.002 24182705

[25] Bhutani G Nasr SH Said SM Sethi S Fervenza FC Morice WG. Hematologic characteristics of proliferative glomerulonephritides with nonorganized monoclonal immunoglobulin deposits. Mayo Clin Proc. (2015) 90:587–96. doi: 10.1016/j.mayocp.2015.01.024 25939936

[26] Nasr SH Markowitz GS Stokes MB Seshan SV Valderrama E Appel GB. Proliferative glomerulonephritis with monoclonal IgG deposits: a distinct entity mimicking immune-complex glomerulonephritis. Kidney Int. (2004) 65:85–96. doi: 10.1111/j.1523-1755.2004.00365.x 14675039

[27] Chauvet S Frémeaux-Bacchi V Petitprez F Karras A Daniel L Burtey S. Treatment of B-cell disorder improves renal outcome of patients with monoclonal gammopathy-associated C3 glomerulopathy. Blood. (2017) 129:1437–47. doi: 10.1182/blood-2016-08-737163 28069603

[28] Zand L Kattah A Fervenza FC Smith RJ Nasr SH Zhang Y. C3 glomerulonephritis associated with monoclonal gammopathy: a case series. Am J Kidney Dis. (2013) 62:506–14. doi: 10.1053/j.ajkd.2013.02.370 PMC4435575 23623956

[29] Martins M Bridoux F Goujon JM Meuleman MS Ribes D Rondeau E. Complement activation and thrombotic microangiopathy associated with monoclonal gammopathy: A national french case series. Am J Kidney Dis. (2022) 80:341–52. doi: 10.1053/j.ajkd.2021.12.014 35217094

[30] Shankar M Anandh U Guditi S. Multiple facets of multiple myeloma in kidney biopsy: A multicenter retrospective study. Indian J Nephrol. (2024) 34:31–6. doi: 10.4103/ijn.ijn_362_22 PMC11003587 38645901

[31] Shankar M Anandh U Guditi S. PARAKID: Navigating the relation between paraproteins and kidney lesions: A multi-center retrospective observational study. Clin Nephrol. (2023) 100:269–74. doi: 10.5414/CN111123 37870264

[32] Borza DB Chedid MF Colon S Lager DJ Leung N Fervenza FC. Recurrent Goodpasture’s disease secondary to a monoclonal IgA1-kappa antibody autoreactive with the alpha1/alpha2 chains of type IV collagen. Am J Kidney Dis. (2005) 45:397–406. doi: 10.1053/j.ajkd.2004.09.029 15685519

[33] Larsen CP Ambuzs JM Bonsib SM Boils CL Cossey LN Messias NC. Membranous-like glomerulopathy with masked IgG kappa deposits. Kidney Int. (2014) 86:154–61. doi: 10.1038/ki.2013.548 24429395

[34] Larsen CP Messias NC Walker PD Fidler ME Cornell LD Hernandez LH. Membranoproliferative glomerulonephritis with masked monotypic immunoglobulin deposits. Kidney Int. (2015) 88:867–73. doi: 10.1038/ki.2015.195 PMC4687465 26154922

[35] Fish R Pinney J Jain P Addison C Jones C Jayawardene S. The incidence of major hemorrhagic complications after renal biopsies in patients with monoclonal gammopathies. Clin J Am Soc Nephrol. (2010) 5:1977–80. doi: 10.2215/CJN.00650110 PMC3001781 20651154

[36] Katzmann JA Kyle RA Benson J Larson DR Snyder MR Lust JA. Screening panels for detection of monoclonal gammopathies. Clin Chem. (2009) 55:1517–22. doi: 10.1373/clinchem.2009.126664 PMC3773468 19520758

[37] Palladini G Russo P Bosoni T Verga L Sarais G Lavatelli F. Identification of amyloidogenic light chains requires the combination of serum-free light chain assay with immunofixation of serum and urine. Clin Chem. (2009) 55:499–504. doi: 10.1373/clinchem.2008.117143 19131635

[38] Gumber R Cohen JB Palmer MB Kobrin SM Vogl DT Wasserstein AG. A clone-directed approach may improve diagnosis and treatment of proliferative glomerulonephritis with monoclonal immunoglobulin deposits. Kidney Int. (2018) 94:199–205. doi: 10.1016/j.kint.2018.02.020 29759418

[39] Cohen C Royer B Javaugue V Szalat R El Karoui K Caulier A. Bortezomib produces high hematological response rates with prolonged renal survival in monoclonal immunoglobulin deposition disease. Kidney Int. (2015) 88:1135–43. doi: 10.1038/ki.2015.201 26176826

[40] Czarnecki PG Lager DJ Leung N Dispenzieri A Cosio FG Fervenza FC. Long-term outcome of kidney transplantation in patients with fibrillary glomerulonephritis or monoclonal gammopathy with fibrillary deposits. Kidney Int. (2009) 75:420–7. doi: 10.1038/ki.2008.577 19037251

[41] Zand L Rajkumar SV Leung N Sethi S El Ters M Fervenza FC. Safety and efficacy of daratumumab in patients with proliferative GN with monoclonal immunoglobulin deposits. J Am Soc Nephrol. (2021) 32:1163–73. doi: 10.1681/ASN.2020101541 PMC8259683 33685975

[42] Zhou H Li M Zeng C Chen Z Zhang T Cheng Z. Efficacy of immunomodulatory drugs in combination with dexamethasone in proliferative glomerulonephritis with monoclonal immunoglobulin deposits. Kidney Int Rep. (2022) 7(10):2166-175. doi: 10.1016/j.ekir.2022.07.009 PMC9546741 36217516

[43] Lin L Chen N. A review on the diagnosis and treatment of proliferative glomerulonephritis with monoclonal immunoglobulin deposits. Int J Gen Med. (2022) 15:8577–82. doi: 10.2147/IJGM.S386733 PMC9760043 36540764

[44] Ravindran A Fervenza FC Smith RJH. Sethi S.C3glomerulopathy associated with monoclonal Ig is a distinct subtype. Kidney Int. (2018) 94:178. 29729982 10.1016/j.kint.2018.01.037 PMC7735221

[45] Ma CX Lacy MQ Rompala JF Dispenzieri A Rajkumar SV Greipp PR. Aquired Fanconi syndrome is an indolent disorder in the absence of over multiple myeloma. Blood. (2004) 104:40. 15010372 10.1182/blood-2003-10-3400

[46] Stokes MB Valeri AM Herlitz L Khan AM Siegel DS Markowitz GS. Light chain proximal tubulopathy: clinical and pathologic characteristics in the modern treatment era. J Am Soc Nephrol. (2016) 27:1555. 26374607 10.1681/ASN.2015020185 PMC4849818

[47] Nasr SH Kudose S Javaugue V Harel S Said SM Pascal V. Pathological characteristics of light chain crystalline podocytopathy. Kidney Int. (2023) 103:616. 36581019 10.1016/j.kint.2022.11.026

[48] Palladini G Dispenzieri A Gertz MA Kumar S Wechalekar A Hawkins PN. New criteria for response to treatment in immunoglobulin light chain amyloidosis based on free light chain measurement and cardiac biomarkers: impact on survival outcomes. J Clin Oncol. (2012) 30:4541–9. doi: 10.1200/JCO.2011.37.7614 23091105

[49] Dispenzieri A Gertz MA Kyle RA Lacy MQ Burritt MF Therneau TM. Serum cardiac troponins and N-terminal pro-brain natriuretic peptide: a staging system for primary systemic amyloidosis. J Clin Oncol. (2004) 22:3751–7. doi: 10.1200/JCO.2004.03.029 15365071

[50] Venner CP Lane T Foard D Rannigan L Gibbs SD Pinney JH. Cyclophosphamide, bortezomib, and dexamethasone therapy in AL amyloidosis is associated with high clonal response rates and prolonged progression-free survival. Blood. (2012) 119:4387–90. doi: 10.1182/blood-2011-10-388462 22331187

[51] Mikhael JR Schuster SR Jimenez-Zepeda VH Bello N Spong J Reeder CB. Cyclophosphamide-bortezomib-dexamethasone (CyBorD) produces rapid and complete hematologic response in patients with AL amyloidosis. Blood. (2012) 119:4391–4. doi: 10.1182/blood-2011-11-390930 PMC3557400 22331188

[52] Wechalekar AD Goodman HJ Lachmann HJ Offer M Hawkins PN Gillmore JD. Safety and efficacy of risk-adapted cyclophosphamide, thalidomide, and dexamethasone in systemic AL amyloidosis. Blood. (2007) 109:457–64. doi: 10.1182/blood-2006-07-035352 16990593

[53] Cibeira MT Sanchorawala V Seldin DC Quillen K Berk JL Dember LM. Outcome of AL amyloidosis after high-dose melphalan and autologous stem cell transplantation: long-term results in a series of 421 patients. Blood. (2011) 118:4346–52. doi: 10.1182/blood-2011-01-330738 PMC3204906 21828140

[54] Cordes S Dispenzieri A Lacy MQ Hayman SR Buadi FK Dingli D. Ten-year survival after autologous stem cell transplantation for immunoglobulin light chain amyloidosis. Cancer. (2012) 118:6105–9. doi: 10.1002/cncr.27660 22707405

[55] Kastritis E Migkou M Gavriatopoulou M Zirogiannis P Hadjikonstantinou V Dimopoulos MA. Treatment of light chain deposition disease with bortezomib and dexamethasone. Haematologica. (2009) 94:300–2. doi: 10.3324/haematol.13548 PMC2635400 19066331

[56] Gozzetti A Guarnieri A Zamagni E Zakharova E Coriu D Bittrich M. Monoclonal gammopathy of renal significance (MGRS): Real-world data on outcomes and prognostic factors. Am J Hematol. (2022) 97:877–84. doi: 10.1002/ajh.26566 PMC9324084 35389534

[57] Kastritis E Palladini G Minnema MC Wechalekar AD Jaccard A Lee HC. Daratumumab-based treatment for immunoglobulin light-chain amyloidosis. N Engl J Med. (2021) 385:46–58. 34192431 10.1056/NEJMoa2028631

[58] Dima D Hughes M Orland M Ullah F Goel U Anwer F. Outcomes of venetoclax-based therapy in patients with t(11;14) light chain amyloidosis after failure of daratumumab-based therapy. Amyloid. (2024) 1–7. 10.1080/13506129.2024.2366806 38956891

[59] Liedtke M Merlini G Landau H Comenzo RL Sanchorawala V Weiss BM. The VITAL amyloidosis study: A randomized, double-blind, placebo controlled, global, phase 3 study of NEOD001 in patients with AL amyloidosis and cardiac dysfunction. Blood. (2016) 128:5690.

[60] Gertz MA Cohen AD Comenzo RL Kastritis E Landau HJ Libby EN. Birtamimab plus standard of care in light-chain amyloidosis: the phase 3 randomized placebo-controlled VITAL trial. Blood. (2023) 142:1208–18. 10.1182/blood.2022019406 PMC10644097 37366170

[61] A Study to Evaluate the Efficacy and Safety of Birtamimab in Mayo Stage IV Patients With AL Amyloidosis (AFFIRM-AL) . Available online at: https://clinicaltrials.gov/study/NCT04973137 (April 14, 2024).

[62] Kyle RA Durie BG Rajkumar SV Landgren O Blade J Merlini G. Monoclonal gammopathy of undetermined significance (MGUS) and smoldering (asymptomatic) multiple myeloma: IMWG consensus perspectives risk factors for progression and guidelines for monitoring and management. Leukemia. (2010) 24:1121–7. 10.1038/leu.2010.60 PMC7020664 20410922

[63] Klomjit N Leung N Fervenza F Sethi S Zand L. Rate and predictors of finding monoclonal gammopathy of renal significance (MGRS) lesions on kidney biopsy in patients with monoclonal gammopathy. J Am Soc Nephrol. (2020) 31:2400–11. doi: 10.1681/ASN.2020010054 PMC7609011 32747354

